# Chronic sleep deficiency and its impact on pain perception in healthy females

**DOI:** 10.1111/jsr.14284

**Published:** 2024-07-07

**Authors:** Shima Rouhi, Natalia Egorova‐Brumley, Amy S. Jordan

**Affiliations:** ^1^ The University of Melbourne Melbourne Victoria Australia

**Keywords:** conditioned pain modulation, experimental pain, sleep deprivation, temporal summation

## Abstract

Acute sleep deprivation in experimental studies has been shown to induce pain hypersensitivity in females. However, the impact of natural sleep deficiency and fluctuations across the week on pain perception remains unclear. A sleep‐monitoring headband and self‐reports were utilized to assess objective and subjective sleep in longer (> 6 hr) and short sleepers (< 6 hr). Pain sensitivity measures including heat, cold, pressure pain thresholds, pain inhibition (conditioned pain modulation) and facilitation (tonic pain summation) were assessed on Mondays and Fridays. Forty‐one healthy young (23.9 ± 0.74 years) women participated. Short sleepers slept on average 2 hr less than longer sleepers (297.9 ± 8.2 min versus 418.5 ± 10.9 min) and experienced impaired pain inhibitory response (mean = −21.14 ± 7.9°C versus mean = 15.39 ± 9.5°C; *p* = 0.005). However, no effect was observed in pain thresholds and pain summation (*p* > 0.05). Furthermore, pain modulatory responses differed between Mondays and Fridays. Chronic sleep deficiency (< 6 hr) compromises pain responses, notably on Mondays. Maintaining a consistent sleep pattern with sufficient sleep (> 6 hr) throughout the week may protect against pain sensitization and the development of chronic pain in females. Further research is needed, especially in patients with chronic pain.

## 1 INTRODUCTION

Chronic pain poses a significant social and economic burden on society due to its disabling nature and high prevalence (Australia, 2019; Economics, 2019; Firoozi & Rouhi, 2020; Rouhi et al., 2020). Among the various comorbidities associated with chronic pain, sleep disturbance is particularly detrimental, and affects over 88% of patients with chronic pain (Sun et al., 2021). The sleep characteristics that are most commonly reported in chronic pain populations are reduced total sleep time (TST), diminished sleep efficiency and an increased frequency of awakenings (Bjurstrom & Irwin, 2016; McCracken & Iverson, 2002; O'Donoghue et al., 2009; Stroemel‐Scheder et al., 2019; Wilson et al., 2022). In addition to being very common, sleep disturbance also increases the prevalence of new‐onset pain conditions (Afolalu et al., 2018). A systematic review of 16 longitudinal studies in pain‐free individuals revealed that a progressive deterioration in sleep quality and quantity over time increased the risk of developing chronic pain conditions by two–threefold; conversely, improved sleep was associated with improved physical functioning, bodily pain and general health using self‐reported health survey (SF‐36; Afolalu et al., 2018; Bjurstrom & Irwin, 2016).

Experimental reductions of sleep in healthy populations that used quantitative sensory testing to measure pain also show a clear relationship, with even minor reductions in sleep leading to heightened pain perception (Eichhorn et al., 2018; Rouhi et al., 2023). Two systematic reviews (Chang et al., 2022; Rouhi et al., 2023) have highlighted the dose‐dependent pain hypersensitivity induced by sleep deprivation in healthy individuals, with total sleep deprivation producing the most significant effect (large), followed by sleep restriction (medium–large) and fragmented sleep (small; Rouhi et al., 2023), all primarily affecting pain thresholds. Moreover, sleep loss impacts various types of pain differentially, with the heat pain threshold being the most affected, followed by pressure and cold pain thresholds (Ramaswamy & Wodehouse, 2021). Studies have also indicated that sleep loss can alter the pain‐modulating pathways in the central nervous system, as measured by conditioned pain modulation (CPM; dampened pain sensation with concurrent painful stimuli; Rouhi et al., 2023) and temporal summation (augmented pain sensation with repeated stimuli). Notably, these sleep‐loss‐related hyperalgesia findings were observed in sleep‐deprived females, with no such effects observed in sleep‐deprived males (Rouhi et al., 2023), albeit with a small effect size. The sex‐related effect of sleep loss on descending pain pathways has been well studied, with research indicating that females demonstrate increased vulnerability, while males do not show significant impact (Eichhorn et al., 2018; Rouhi et al., 2023; Smith Jr et al., 2018).

Experimental studies such as these have induced sleep deprivation of up to 3 nights and then measured pain, such that the reported hyperalgesia is mainly attributed to the acute and immediate effect of sleep loss (Matre et al., 2016; Schuh‐Hofer et al., 2013; Smith et al., 2007). Whether similar effects would be observed in naturally occurring sleep deficiency, defined as regularly getting less than 6 hr of sleep per night, is less clear (Campbell et al., 2011). One study that used self‐reported sleep data found that a sleep duration of less than 6.5 hr was associated with increased secondary hyperalgesia in healthy individuals as compared with those who slept more than 6.5 hr (Campbell et al., 2011). However, this study was based on subjective sleep data and only measured heat pain. Given the steady decline in average nocturnal sleep duration and the high prevalence of chronic sleep deficiency, which affects up to 53.3% of individuals (Ford et al., 2015; Matsumoto & Chin, 2019), it is necessary to explore how chronic sleep deficiency may influence other aspects of pain perception and with objective sleep assessment.

The present study therefore aimed to investigate how natural variations in TST affect pain perception in women. Specifically, we wanted to understand how consistently getting less than 6 hr of sleep over a week (without any experimental manipulation of sleep) could impact pain perception. We hypothesized that individuals with short sleep duration would exhibit higher pain sensitivity than those with longer sleep duration. Furthermore, we hypothesized that pain hypersensitivity would be more pronounced on Mondays (following the weekends when irregular sleep times may be observed) than on Fridays (following weekdays with more regular sleep timing) for both short‐ and long‐sleeping females. To eliminate the potential sex‐moderating effect of sleep loss on pain perception, the study included only healthy females (Rouhi et al., 2023).

## 2 MATERIALS AND METHODS

### 2.1 Study design and recruitment procedure

**FIGURE 1:**
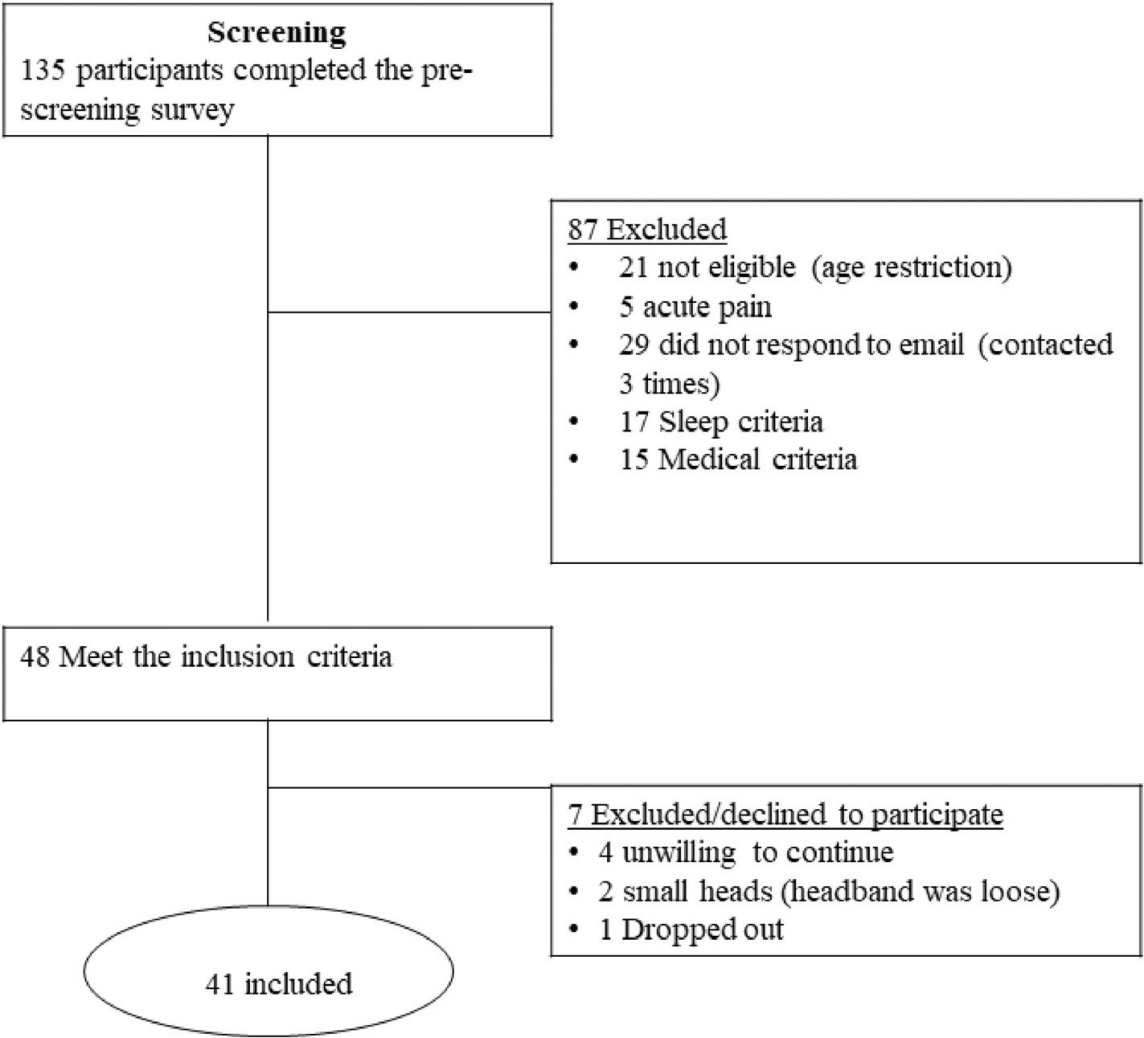
Prisma flow chart. Study consort diagram. Following the pre‐screening survey, including sleep pattern inquiries, general health and medical history, and demographic details, eligible participants were grouped into short and longer sleepers based on their self‐report sleep quantity to participate in the study for the entire week starting on Tuesday or Friday. The numbers of participants excluded from the study, before or after pre‐screening assessments, is detailed along with the reasons for exclusion.

The study received ethical approval from the Human Research Ethics Committees (reference number: 2023‐25168‐43267‐6) at the University of Melbourne. Participants identified as healthy and pain‐free were recruited through advertising on university platforms and distributing flyers across the University of Melbourne Campus. Before participating, all recruited individuals were presented with a consent form. Upon providing consent, participants were required to complete baseline questionnaires that included a general health questionnaire (inquiring into drug, caffeine, alcohol consumption, medical or psychological history, and smoking status), demographic information (such as age, ethnicity and socio‐economic status), and the Pittsburgh Sleep Quality Index (PSQI) to assess eligibility. Based on their self‐reports, participants were grouped into short sleepers (6 hr and fewer) and longer sleepers (more than 6 hr). After study completion, participants were reimbursed AUD 120 for their time. Further information regarding participant flow and study procedures are provided in the Prisma flow chart (Figure 1).

#### 2.1.1 Inclusion and exclusion criteria

##### Inclusion criteria

Eligible individuals were:

- females aged between 18 and 35 years. The age restriction was imposed to minimize age‐related effects on pain sensitivity (Eltumi & Tashani, 2017), particularly in thermal pain threshold (Edwards & Fillingim, 2001);
- free from severe medical or psychiatric conditions;
- free from sleep disorders (self‐reported);
- non‐smokers and non‐nicotine users;
- low caffeine intake consumes less than two cups or an equivalent daily.

##### Exclusion criteria

Individuals with the following conditions were excluded:

- chronic or acute pain;
- significant medical, cardiorespiratory, neurological or psychiatric condition;
- psychotic disorder or recurrent major depression;
- a lifetime history of alcohol or substance abuse;
- use of antidepressant or other medications that could impact sleep or pain within the last 6 months.

#### 2.1.2 Sample size estimation

We conducted an a priori power analysis using G*Power 3.1.33 to estimate the sample size needed for assessing pain differences over 2 days (Monday and Friday) between groups (short versus longer sleepers) using repeated‐measures ANOVA with a within‐between interaction. The analysis aimed to detect a small effect size (*f* = 0.25) with a critical *F*‐value of 4.1. Based on this analysis, it was determined that a sample size of *N* = 34 participants (17 in each group) would be required to observe a significant effect at *α* = 0.05 with 80% power.

#### 2.1.3 Procedure

**FIGURE 2:**
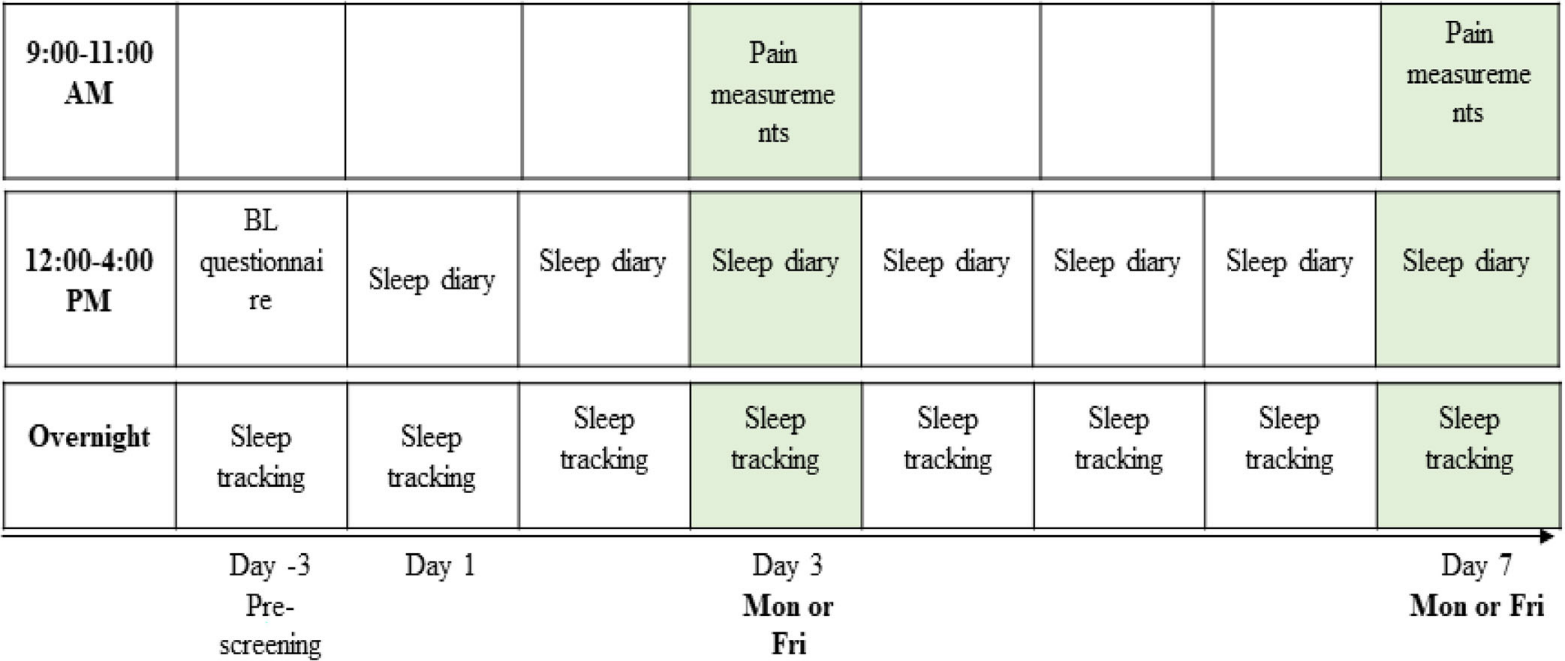
Study design and flow. Participants were pre‐screened for up to 3 nights to adjust to the headband, Sleep tracking: sleep data were collected using the Dreem headband; pain measurements: quantitative sensory testing: heat and pressure thresholds, conditioned pain modulation (CPM) and temporal summation; Day 3 and Day 7 varied based on participant intake, either on Monday or Friday, to maintain order balance. Sleep diaries: self‐reported sleep questionnaires. Both short and longer sleepers followed the same procedure. BL, baseline. This figure has been obtained from our previous experiment (currently under review).

The current study aimed to investigate the relationship between sleep duration and pain perception by measuring sleep at participants' homes throughout the week, utilizing both objective measures (DREEM headband, DREEM 2 or DREEM 3; Beacon Biosignals, Boston, MA, USA; Arnal et al., 2020) and subjective measures (sleep diary questionnaire). Additionally, two identical pain sensitivity testing sessions were conducted in the pain laboratory at the University of Melbourne to assess various aspects of pain processing. These sessions were consistently held on Mondays (following weekend sleep) and Fridays (following weekday sleep) between 09:00 hours and 11:00 hours. Participants initiated sleep monitoring on Friday evening or Tuesday evening (approximately counterbalanced but adjusted based on illness or availability to attend the laboratory) to minimize the likelihood of order effects influencing results. The study design (Figure 2) provides further details.

##### Experimental pain testing

The pain testing procedure was identical for both groups. During each testing session, the pain assessments were administered in a predetermined order, starting with heat pain threshold, then cold pain threshold, pressure pain threshold (PPT), temporal summation of heat pain, and concluding with CPM. In order to mitigate the order (Karmann et al., 2018) effects of previous pain, we began the process with pain threshold assessments (consisting of slightly painful stimuli). We subsequently proceeded to more long‐lasting painful stimuli (temporal summation of heat pain). Finally, we concluded the testing session with CPM, which involved holding one's hand in an ice bucket as the conditioning stimulus. A 2‐min interval was observed between each measurement to minimize any carry‐over effects. Each pain testing session took less than 1 hr.

### 2.2 Questionnaires and surveys

#### 2.2.1 Pittsburgh Sleep Quality Index (PSQI)

This sleep questionnaire, consisting of 19 well‐validated questions (Buysse et al., 1989), was administered to assess sleep quality. A total score of ≤ 5 indicates good sleep quality, and all sub‐dimensions derived from the PSQI were utilized for subsequent assessments.

#### 2.2.2 Epworth Sleepiness Scale (ESS)

The ESS is an eight‐item questionnaire that is used to evaluate daytime sleepiness (Johns, 1991). The participants are required to assess their likelihood of dozing off or falling asleep in different scenarios, using a scale ranging from 0 (would never doze) to 3 (high chance of dozing). This questionnaire has been widely used in sleep research and clinical practice to assess the level of daytime sleepiness experienced by individuals.

#### 2.2.3 Beck Depression Inventory (BDI)

The BDI is a self‐report questionnaire that comprises 21 items designed to assess depressive symptoms in both normal and clinical populations (Jackson‐Koku, 2016). Each question employs a four‐point scale, ranging from 0 (no symptom) to 3 (severe symptoms). The total score is calculated by summing all items, resulting in a range from 0 to 63. Interpretation of scores categorizes them as follows: 0–13 as minimal; 14–19 as mild; 20–28 as moderate; and 29 and above as indicative of severe depression.

### 2.3 Evaluation of pain‐related metrics

#### 2.3.1 Equipment

The thermal pain thresholds (heat and cold) were assessed using the Medoc Pathway (MEDOC, Israel; Rolke et al., 2006), and have been previously utilized in several studies (Eichhorn et al., 2018; Schuh‐Hofer et al., 2013). The device is equipped with a contact probe that has a surface area of 9 cm^2^, and operates within a cut‐off temperature range of 0–50°C. In addition, for PPT evaluation, a Medoc Algomed pressure algometer with a probe diameter of 1.0 cm^2^ was used.

#### 2.3.2 Thermal pain thresholds (heat and cold)

The baseline temperature was set to 32°C. During the experiment, the temperature of the thermode increased/decreased at a rate of 0.5°C per second until the participant perceived the stimuli as painful. Participants were instructed to press a response button when the sensation transitioned from noticeable to painful (Rødsjø, 2020). The contact probe was positioned on the dorsal part of the forearm 10 cm from the elbow crest on the non‐dominant hand. Finally, the cold and heat pain thresholds were quantified by calculating the arithmetic mean of the three measurements.

#### 2.3.3 Pressure pain threshold (PPT)

We used a Medoc Algomed pressure algometer with a probe diameter of 1.0 cm^2^ to measure PPT. The assessment of the PPT involved a ramp‐up rate of 50 kPa s^−1^, with participants instructed to press a response button as soon as the sensation became painful (Eichhorn et al., 2018). After each button was pressed, the probe was removed, and the pressure was returned to zero before proceeding with the subsequent evaluation. The non‐dominant hand's thenar eminence was the placement site for the probe, and 8 s separated each stimulus. The PPT was determined by calculating the average of three measurements (PPTpre).

#### 2.3.4 Tonic heat pain summation

To measure tonic heat pain summation, we employed a method similar to that used to determine the heat pain threshold. We individually calibrated the painful stimuli based on the “pain‐6” value. Pain 6 was measured by applying five different temperatures (43, 44, 45, 46 and 47°C), each lasting for 7 s. Participants were then asked to rate the level of pain experienced on a numerical rating scale (NRS) ranging from 0 to 10. The temperature corresponding to a pain level of 6 was selected and applied for 60 s, with participants rating their pain levels every 10 s, beginning at 15 s. Any participant who found the heat pain at 43°C intolerable was excluded from the study (Matre et al., 2016; Rødsjø, 2020). The extent of tonic pain summation was calculated by measuring the absolute difference in pain scores between the last and first pain ratings. Larger pain summation values indicate more pronounced pain facilitation, while negative values suggest reduced pain perception.

#### 2.3.5 Conditioned pain modulation (CPM)

After conducting the assessment of PPT on the non‐dominant hand, the participants were instructed to submerge their dominant hand in an ice water tank (maintained between 0 and 2°C). Temperature checks were conducted using a thermometer both before and during the test. They were requested to hold their hand in the cold water (Eichhorn et al., 2018) while spreading out their fingers for as long as they could tolerate, up to a maximum of 2 min, which served as the conditioning stimulus. Following the withdrawal of their hand from the ice water, participants rated their pain using a NRS. The duration, in seconds, of the cold pain tolerance was recorded. Immediately after removing their hand from the ice water, the PPT was re‐evaluated (PPTpost). The effectiveness of CPM was determined by calculating the difference between these PPT values, with positive values indicating effective pain inhibition and negative values indicating pain facilitation as per the protocols of Karmann et al. (2018) and Stroemel‐Scheder et al. (2019).

### 2.4 Evaluation of sleep‐related metrics

#### 2.4.1 Procedure

In the post‐screening phase, participants were instructed to wear the DREEM headband for at least 3 nights before each pain testing session and for up to 12 days in total. Most participants wore the headband for a total of 7 nights, with 3 nights before each pain testing session. However, due to scheduling changes, six participants had to wear the headband for more than 7 nights to meet the requirement. Among these six, four participants wore the headband for up to 12 days. Concurrently, they were required to complete a daily sleep diary each morning.

##### DREEM headband

Both macro‐ and micro‐structural sleep parameters were monitored using the DREEM headband. Macro‐structural sleep parameters included TST, sleep‐onset latency (SOL), wake after sleep onset, bedtime, and wake‐up time, whereas micro‐structural sleep parameters encompassed sleep stages such as non‐rapid eye movement (NREM) (N1, N2, N3) stages and rapid eye movement (REM). The DREEM headband has been validated to have a comparable level of accuracy to polysomnography (PSG) data, achieving an overall sleep staging accuracy of 83.5% compared with 86.4% with PSG. This makes the DREEM headband a reliable alternative for sleep monitoring (Arnal et al., 2020).

##### Sleep diary

The sleep diary questionnaire covered inquiries regarding tiredness, subjective sleep patterns, bedtime, wake‐up time, number of naps, and mood and alertness levels. The initial 10 items of the sleep diary were adapted from the Chalder Fatigue Scale, a tool for quantifying fatigue levels (Chalder et al., 1993). Afterward, other responses, such as time in bed and SOL, were analysed individually. Sleep quality, mood and alertness were quantified using a NRS ranging from 0 to 10, where a higher rating indicated better sleep quality, elevated mood and increased alertness.

### 2.5 Statistical analysis

SPSS version 22 (IBM, Armonk, NY, USA) with a significance level set at *p* < 0.05 was used for statistical analyses. The data are presented as mean and standard deviation. The estimated marginal means ± SEMs are used to represent the data in graphical format. The normality of the data was determined through the Shapiro–Wilk test of normality, supplemented by visual inspections of scatter plots and histogram plots of residuals. Any instances of data non‐normality led to identifying outliers via initial visual inspection of scatter plots. Following this, raw data were transformed to Log10 or ln if normality remained unattained. Non‐parametric tests were applied if necessary, including the Friedman test for repeated measures and the Wilcoxon‐rank test for the non‐parametric *t*‐test.

A repeated‐measures General Linear Model (GLM) was employed to assess sleep variations across the week between individuals grouped as short and longer sleepers, while also investigating the effect of day of the week. Sleep variations were treated as within‐subject factors across the week, with group differences considered as between‐subject factors. The GLM included analyses of intercept and Group effects using multivariate tests. The assumption of sphericity was assessed using Mauchly's Test of Sphericity, and corrections were applied using the Greenhouse–Geisser method if the assumption was violated (*p* < 0.05). Levene's test of equality of error variances was used to evaluate the equality of error variances of the dependent variables.

The GLM with intercept and within‐subject factors (Day of Week) was utilized to conduct multivariate tests, examining differences in pain perception between the groups on both Monday and Friday (Group × Day of the Week). Heat, cold, pressure, pain summation, and pain inhibition were each entered into separate models as within‐subject factors, with groups (short versus longer sleepers) included as between‐subject factors. Box's test of equality of covariance matrices was performed to assess whether the dependent variables were equal across the groups. To control for multiple testing, pairwise comparisons were conducted using the Bonferroni correction with a significance level set at 0.05, and all results have been reported after this adjustment.

## 3 RESULTS

### 3.1 Participants' characteristics

The study included 41 female participants with a young age (longer sleepers: 24.8 ± 5.3 years versus short sleepers: 23.1 ± 3.9 years; *p* = 0.13) and a normal body mass index (longer sleepers: 23.4 ± 2.3 kg m^−2^ versus short sleepers: 23 ± 5.8 kg m^−2^; *p* = 0.37). The PSQI global score indicated poor sleep quality on average (longer sleepers: 7 ± 3.5 versus short sleepers: 8.2 ± 2.7; *p* = 0.12), and the ESS score suggested mild daytime sleepiness (longer sleepers: 9 ± 6 versus short sleepers: 9.5 ± 4; *p* = 0.38), short sleepers exhibited higher scores in both scales, suggesting a trend toward heightened perceived sleepiness in this group. Further information is provided in the supplementary file (Table S1).

Both longer and short sleepers fell within the minimal or no depression range in the BDI score (longer sleepers: 9.5 ± 6.9 versus short sleepers: 11.5 ± 8.8; *p* = 0.21).

Most of the participants (92.7%) were right‐handed, while only three individuals (7.3%) were left‐handed. The majority identified as Asian (70.7%), followed by White (19.5%), and Black or African American (9.8%). The first pain testing session was conducted on Monday for 20 participants (48.8%), while 21 participants (51.2%) had their first pain testing session on Friday.

### 3.2 Weekly sleep variation between longer versus short sleepers

#### 3.2.1 Objective sleep measurements

**FIGURE 3:**
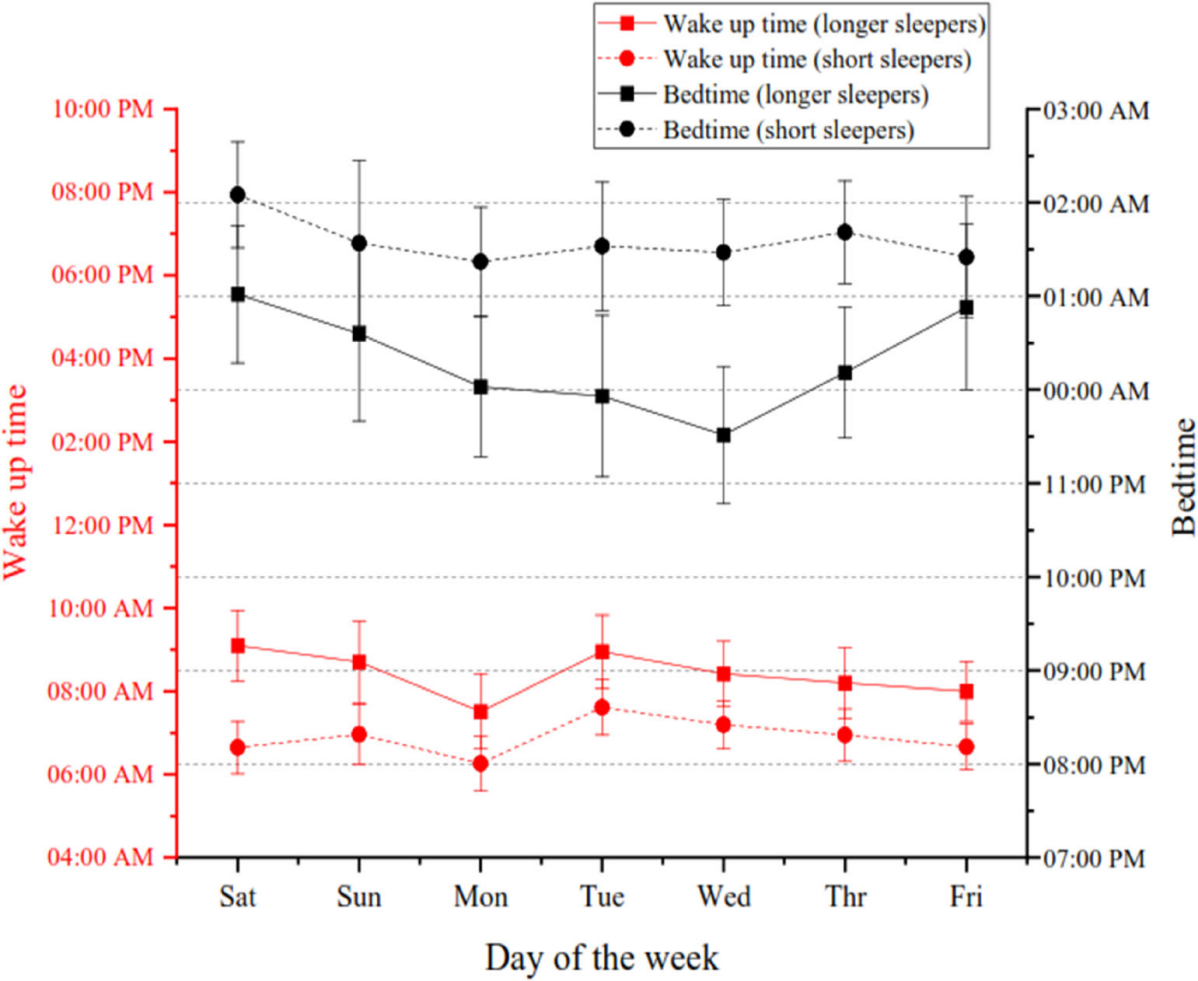
Bedtime and wake‐up times for longer sleepers versus short sleepers. Means and standard errors for bedtimes and wake‐up times across the week, short sleepers are represented in dashed lines and longer sleepers are represented in solid lines.

Longer sleepers slept on an average 2 hr more than short sleepers (mean difference = 120.56 ± 13.7 min; 95% confidence interval [CI] = 92.7–148.3; *p* < 0.001; Figure 3; Table S3). Short sleepers went to bed later (mean difference = 1.15 ± 0.50 hr; 95% CI = 0.081–2.14; *p* = 0.035; Figure 3) but woke up more than an hour earlier (mean difference = −1.59 ± 0.50 hr; 95% CI = −2.62 to −0.55; *p* = 0.004; Figure 3) than longer sleepers. The duration of all sleep stages was significantly reduced in short sleepers compared with longer sleepers (*p* < 0.05). Longer and short sleepers did not differ in sleep efficacy (mean difference = 0.068 ± 2.01; 95% CI = −4.02 to 4.15; *p* = 0.97) and SOL (mean difference = 9.78 ± 5.46 min; 95% CI = −1.31 to 20.87; *p* = 0.082; *p* = 0.08). For other sleep characteristics, please refer to Table S3 and Figures S1–S6 of the supplementary file.

#### 3.2.2 Subjective sleep measurements

Table S4 provides descriptive data for all sleep parameters. Results indicate that short sleepers reported going to bed at least 1 hr later than longer sleepers (mean difference = 1.0 ± 0.49 hr; 95% CI = 0.011–2; *p* = 0.048), but woke up an hour earlier than longer sleepers (mean difference = −1.24 ± 0.57 hr; 95% CI = −2.4 to −0.078; *p* = 0.037). Other than having a delayed bedtime and early wake‐up time, short sleepers did not differ from longer sleepers in perceived SOL (mean difference = −1.39 ± 4.6 min; 95% CI = −10.8 to 8; *p* = 0.76; *p* = 0.76). Sleep quality was slightly higher in longer sleepers (mean difference = 1.01 ± 0.53; 95% CI = −0.071 to 2; *p* = 0.06), but lower wakefulness after falling asleep (mean difference = −1.53 ± 3.07; 95% CI = −7.75 to 4.6; *p* = 0.6), albeit no statistically significant difference.

### 3.3 Comparing sleep patterns on the night before pain testing

#### 3.3.1 Longer versus short sleepers

For the detailed sleep characteristics on the night before pain testing see Table S3. Longer sleepers slept on average more than 1 hr more than short sleepers (mean difference = 88.9 ± 21.33 min; 95% CI = 45.8–132.15; *p* < 0.001), characterized by earlier bedtime (mean difference = −0.65 ± 0.54 hr; 95% CI = −1.7 to 0.45; *p* = 0.23) and later wake‐up time (mean difference = 0.80 ± 0.51 hr; 95% CI = −0.22 to 1.8; *p* = 0.12), albeit no statistically significant difference in bedtime and wake‐up time between groups. Time taken to fall asleep was comparable between longer and short sleepers (mean difference = 5.4 ± 4.5 min; 95% CI = −3.8 to 14.7; *p* = 0.24). Moreover, all sleep stages were reduced in short sleepers (*p* < 0.05), with the least reduction observed in the N3 sleep stage (mean difference = −17.61 ± 9.5 min; 95% CI = −36.85 to 1.6; *p* = 0.07). Short sleepers showed fewer awakenings than longer sleepers (mean difference = −5.3 ± 1.6; 95% CI = −8.7 to −2; *p* = 0.002). Despite the sleep constraint in short sleepers, the distribution of sleep stages did not differ between longer sleepers and short sleepers (*p* > 0.05). In sum, short sleepers slept less on average compared with longer sleepers, but exhibited a consolidated sleep pattern with a comparable duration of SOL and fewer awakenings. This resulted in an equivalent amount of deep sleep (N3 sleep stage) as observed in longer sleepers.

#### 3.3.2 Monday versus Friday

Detailed sleep characteristics are reported in Table S3. All sleep parameters remained consistent between Monday and Friday within subjects, except for SOL, which showed a significant difference (mean difference = −10.3 ± 4.4 min; 95% CI = −19.2 to −1.3; *p* = 0.02), indicating that both groups had shorter SOL on Monday compared with Friday.

### 3.4 Comparing pain changes

#### 3.4.1 Pain thresholds

##### Longer versus short sleepers

No significant difference was observed in the pain thresholds between longer and short sleepers for heat (mean difference = 0.43 ± 0.76°C; 95% CI = −1.1 to 1.9; *p* = 0.57; Figure S7), cold (mean difference = −0.85 ± 1.05°C; 95% CI = −2.9 to 1.2; *p* = 0.42; Figure S7) and pressure pain stimuli (mean difference = −7.64 ± 46.70 kPa; 95% CI = −102.1 to 86.8; *p* = 0.87; Figure S7).

##### Monday versus Friday

There were no significant within‐subject differences between Monday and Friday for heat (mean difference = −0.26 ± 0.36°C; 95% CI = −0.99 to 0.476; *p* = 0.47), cold (mean difference = −0.85 ± 1°C; 95% CI = −2.9 to 1.2; *p* = 0.42) and pressure pain stimuli (mean difference = −11.7 to 13.5 kPa; 95% CI = −39.1 to 15.7; *p* = 0.39).

#### 3.4.2 Tonic pain summation

##### Longer versus short sleepers

**FIGURE 4:**
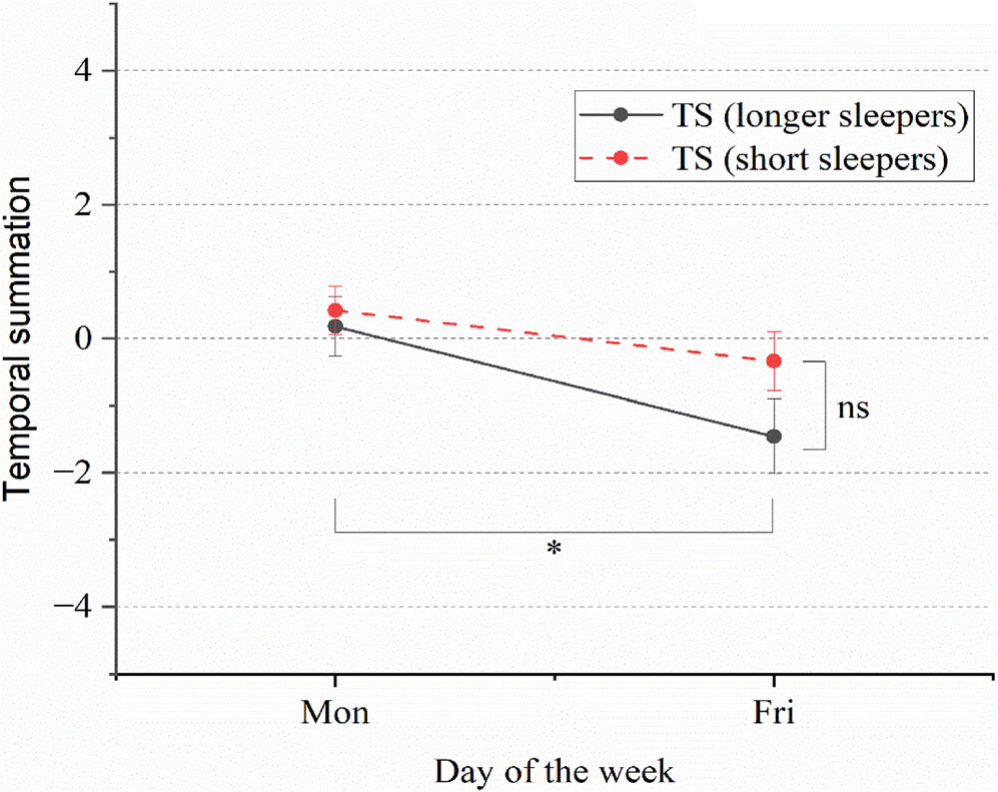
Tonic pain summation in longer versus short sleepers. Means and standard errors for temporal summation on Monday and Friday in short and longer sleepers. Solid and dashed lines, respectively, represent longer and short sleepers. In the figure, *y*‐axis for pain ratings to a given painful stimuli (difference from the final [55 s] to the initial painful stimuli [15 s]), and *x*‐axis represents the day of the week. Positive values demonstrated increased pain perception, while negative values showed decreased pain perception.

No significant difference was observed in the tonic pain summation between longer and short sleepers (mean difference = −0.83 ± 0.52; 95% CI = −1.8 to 0.23; *p* = 0.12; Figure 4). Although not statistically significant, longer sleepers tended to experience decreased heat pain perception (negative value), while short sleepers displayed increased heat pain perception (positive value). Detailed information is provided in Figure S8.

##### Monday versus Friday

Significant within‐subject effects were observed between Monday and Friday (mean difference = 1.1 ± 0.37; 95% CI = 0.38–1.9; *p* = 0.004; Figure 4). Pain summation (positive value) and overall increased pain sensation was observed on Monday, whereas decreased pain sensation (negative value) was observed on Friday.

#### 3.4.3 Conditioned pain modulation (CPM)

##### Longer versus short sleepers

**FIGURE 5:**
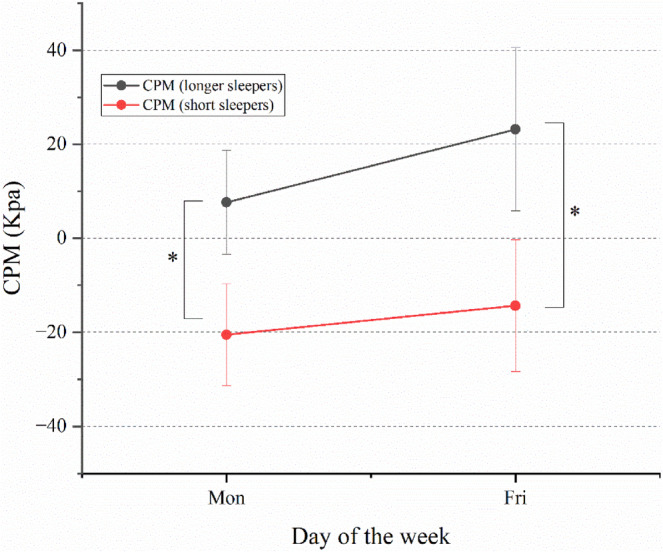
Conditioned pain modulation (CPM) in longer sleepers versus short sleepers. CPM between Monday and Friday in longer and short sleepers. The *y*‐axis represents the CPM value, where positive values indicate effective pain inhibitory capacity, while negative values suggest an impaired pain inhibitory response.

A significant difference was observed in the CPM between longer and short sleepers (mean difference = 36.5 ± 12.3 kPa; 95% CI = 11.4–61.6; *p* = 0.005; Figure 5). Pain inhibitory response was effective (positive value) in longer sleepers but impaired (negative value) in short sleepers.

##### Monday versus Friday

No within‐subject effects were observed between Monday and Friday (mean difference = −7.15 ± 14.6; 95% CI = −36.8 to 22.5; *p* = 0.62; Figure 5). The direction of effect showed negative value (indicating diminished pain‐inhibitory response on Monday) but positive value (indicating an effective response on Friday) for both groups, but not statistically significant.

### 3.5 Comparing emotional states: alertness, mood, fatigue

#### 3.5.1 Longer versus short sleepers

Detailed information can be found in Table S4. No significant difference was observed between longer and short sleepers, including alertness (mean difference = 0.76 ± 0.52; 95% CI = −0.30 to 1.83; *p* = 0.15), mood (mean difference = 0.46 ± 0.49; 95% CI = −0.54 to 1.46; *p* = 0.36; *p* = 0.77) and fatigue (mean difference = 0.32 ± 2; 95% CI = −3.8 to 4.5; *p* = 0.87).

#### 3.5.2 Monday versus Friday

No within‐subject effects were observed between Monday and Friday for level of alertness (mean difference = 0.14 ± 0.27; 95% CI 14;= −0.41 to 0.71; *p* = 0.60), mood (mean difference = −0.25 ± 0.27; 95% CI = −0.81 to 30; *p* = 0.36) and fatigue (mean difference = −0.56 ± 1.2; 95% CI = −3.01 to 1.8; *p* = 0.64).

### 3.6 Exploratory analyses

A significant interaction between group and day was observed for level of alertness (longer sleepers: Monday = 6 ± 0.52 versus Friday = 5.2 ± 0.54; short sleepers: Monday = 5 ± 0.41 versus Friday = 5.5 ± 43; *F*_1,39_ = 5.6; power = 0.64; *p* = 0.02), and fatigue (longer sleepers: Monday = 8.3 ± 2 versus Friday = 12 ± 1.8; short sleepers: Monday = 11.6 ± 1.6 versus Friday = 9 ± 1.4; *F*_1,39_ = 6.6; power = 0.71; *p* = 0.014). Results showed that longer sleepers displayed a higher level of alertness on Monday but diminished toward the end of the week (on Friday) compared with short sleepers. Likewise, long sleepers had lower levels of fatigue on Monday but gradually decreased toward the end of the week (on Friday), while short sleepers displayed higher levels of fatigue on Monday. For more information, please refer to Table S4.

## 4 DISCUSSION

The study investigated the impact of habitually short sleep duration (< 6 hr) on pain perception. Contrary to the initial hypothesis, curtailed sleep did not affect basic pain thresholds (heat, cold and pressure) or pain facilitation (tonic pain summation). However, reduced sleep quantity impaired the CPM aspect of pain processing (inhibitory response). This suggests that non‐pathological sleep curtailment may heighten pain sensitivity by affecting dynamic pain inhibition.

### 4.1 Sleep characteristics and variations across a week

Longer sleepers displayed an expected irregular sleep pattern, with a notably delayed bedtime on Friday night (01:33 ± 0.48 hours) and the latest wake‐up on Saturday morning (09:05 ± 0.51 hours). This sleep pattern aligns with prior research on young adults (Crowley & Carskadon, 2010; Taylor et al., 2008; Valdez et al., 1996), illustrating that university students undergo a shift to irregular sleep timing during the transition from weekdays to weekends. This shift is characterized by a later bedtime and waking time compared with school days (Crowley & Carskadon, 2010; Taylor et al., 2008; Valdez et al., 1996). Consistent with the current findings in longer sleepers, previous research has reported an average sleep duration of 6.9 ± 1.3 hr on weeknights for young adults, increasing to 8 hr over weekends (Chhangani et al., 2009). The increase in TST over the weekend was not as marked in our population, perhaps reflecting a high percentage of international students who have part‐time jobs on weekends.

Short sleepers consistently displayed a reduction in sleep duration, averaging 5 hr and 37 min of sleep, with a delayed sleep phase (01:35 ± 0.35 hours) and an early wake‐up time (06:54 ± 30 hours) throughout the week. In contrast to the longer sleepers, short sleepers did not exhibit irregular sleep patterns or compensate for sleep debt over weekends; rather, their sleep duration was continued to be constrained on weekends. Notably, Friday night appeared as the second shortest night of the week, marked by the latest bedtime and earliest waking time. This suggests an absence of deliberate efforts by short sleepers to offset accumulated sleep debt. The observed pattern might underscore the alignment between desire and actual sleep among short sleepers, indicating self‐perceived sleep sufficiency.

Despite the absence of perceived deficiency in sleep in short sleepers (low ESS, PSQI and lack of weekend rebound effects), we observed impaired pain processing in the shorter sleeper group, along with a day of the week interaction (with more impairment in pain observed on Monday versus Friday). The observed dysfunction in inhibitory pathways suggests that prolonged reduction in sleep duration may pose a risk for developing future pain. This finding aligns with a systematic review by Watson et al. (2015), emphasizing that sleeping less than 6 hr is linked to elevated pain symptoms and spontaneous pain (Watson et al., 2015). Notably, more severe forms of sleep deficiency, 5 hr or less of sleep, have been associated with heightened pain perception across all experimental studies (Tamakoshi & Ohno, 2004).

While sleep‐loss‐related hyperalgesia has been largely reported in experimental studies (Edwards et al., 2008; Rouhi et al., 2023), this paper contributes additional support to the hyperalgesic effect of naturally occurring sleep deficiency. Although the bidirectionality of the sleep–pain interaction has been well established (Haack et al., 2020), particularly in females (Smith et al., 2007), the mechanism by which sleep deficiency leads to this hypersensitivity is still under investigation. The current study revealed that even though short sleepers perceived their sleep as sufficient, as evidenced by the absence of sleep debt compensation over the weekends and the same level of sleepiness between groups, they still exhibited dysfunctional pain inhibitory pathways. These results suggest that pain augmentation in response to sleep loss may extend beyond self‐perceived sleep sufficiency.

A recent systematic review has suggested that sleep deficiency contributes to chronic pain pathogenesis through various potential pathways, including the opioid system, the hypothalamus–pituitary–adrenal (HPA) axis, and inflammatory biomarkers (Haack et al., 2020). Patients with chronic pain exhibit reduced mu‐opioid receptor transmission, causing pain hypersensitivity through endogenous opioid peptide release. Sleep‐deprived rodents display a dysfunctional opioid system, linking inadequate sleep to hyperalgesia via impaired endogenous pain inhibition (Haack et al., 2020). Another potential mechanism involves the HPA axis, influencing nociception by releasing cortisol and inhibiting proinflammatory cytokines. An imbalanced HPA axis, observed in patients with chronic pain, serves as a marker for chronic pain development. Prolonged sleep deficiency, especially in patients with insomnia, contributes to a hyperactive HPA axis, heightening pain sensitivity (Haack et al., 2020). Alternatively, inflammatory biomarkers, including interleukin‐1, interleukin‐6, tumour‐necrosis factor‐alpha and prostaglandins (Fang et al., 2023; Haack et al., 2020), may contribute to increased pain sensitivity in chronic sleep deficiency. These markers are associated with activating pain neurons, contributing to chronic pain onset. Interleukin‐6, produced by immune cells, plays a role in pain augmentation, while blocking it has been shown to reduce pain sensitivity. Sleep deprivation has been attributed to higher prostaglandin levels in animals and increased urinary prostaglandin E2 metabolites in humans, indicating the involvement of prostaglandin E2 in sleep deficiency‐induced pain hypersensitivity (Haack et al., 2020). A study on 18 healthy individuals (six females, 12 males) found that consistent sleep restriction to 4 hr per night over 10 nights resulted in increased interleukin‐6 plasma levels (Haack et al., 2007) and a trend to increase C‐reactive protein levels. Elevated interleukin‐6 levels were associated with reduced NREM sleep. In the current study, short sleepers had just over an hour less NREM sleep compared with longer sleepers. So, it is plausible that chronic sleep deficiency, mediated by the above‐mentioned underlying mechanisms (Haack et al., 2007), results in heightened pain sensitivity. While the underlying mechanisms mentioned may have contributed to the observed compromised pain inhibitory pathways, these factors have not been studied in the current study and require further investigation.

Pain thresholds and tonic pain summation did not differ between longer and short sleepers, suggesting they are more stable and resistant to long‐term sleep changes. Pain thresholds represent the balance between upregulating pain‐facilitatory and downregulating pain‐inhibitory responses at the point where a stimulus is perceived as painful (Bar‐Shalita et al., 2019; Lautenbacher, 2012; Stroemel‐Scheder & Lautenbacher, 2022). Therefore, they reflect the overall balance of an array rather than changes within the system (Bar‐Shalita et al., 2019; Lautenbacher, 2012; Stroemel‐Scheder & Lautenbacher, 2022). In contrast, dynamic pain processing such as CPM reflects any deficiency in the effectiveness of pain inhibitory pathways to suppress pain in the central nervous system (Stroemel‐Scheder & Lautenbacher, 2022).

### 4.2 Strength and limitations

The strength of the current study lies in strictly excluding sex and age as moderating (Rouhi et al., 2023) factors on pain perception, achieved by exclusively including young females (18–35 years). It is recommended that future studies directly compare males and females to investigate the differences in pain perception under sleep loss conditions. The study also employed both objective and subjective sleep measurements throughout the entire week, and implemented counterbalanced pain testing sessions. However, there are limitations that may impact the results and generalizability, as findings may not be extended to male participants and older adults. Replicating this study in an older population is recommended, as age influences CPM (Grashorn et al., 2013), with pain‐inhibitory responses typically decreasing with age (Grashorn et al., 2013). Pain testing sessions were conducted between 09:00 hours and 11:00 hours to mitigate potential circadian effects on pain sensitivity (Hagenauer et al., 2017), but this timing choice might influence the results. Last but not least, the study did not control for menstrual cycle effects (Wilson et al., 2013), which could potentially influence pain perception and sleep. Therefore, future research should consider testing participants during the same menstrual cycle phase to account for this factor.

## 5 CONCLUSION

The findings of this study underscore the significance of sleep quantity, as our results indicated that having less than 6 hr of sleep was associated with impaired pain inhibition, as measured by CPM, regardless of the day of the week. Interestingly, on Mondays, when participants transitioned from the weekend to the workweek, the impact of sleep deficiency on pain modulatory pathways was more noticeable compared with Fridays. As the exploratory analyses showed, despite the common belief that people feel refreshed on Mondays after a weekend's rest, sleep‐deprived individuals reported higher levels of fatigue and lower levels of alertness on this day. However, this effect diminished toward the end of the workweek. These findings suggest that maintaining a consistent sleep pattern, with sufficient sleep (> 6 hr) throughout the week, like that of longer sleepers during working days, may serve as a protective measure against pain sensitization in pain‐free females. Given that sleep is a modifiable factor following a regular and adequate sleep pattern throughout the week may assist in preventing hyperalgesia, particularly on Mondays.

## AUTHOR CONTRIBUTIONS

**Shima Rouhi:** Conceptualization; investigation; writing – original draft; methodology; validation; visualization; writing – review and editing; formal analysis; project administration; data curation. **Natalia Egorova‐Brumley:** Conceptualization; investigation; funding acquisition; supervision; resources; project administration; writing – review and editing; methodology; validation; visualization. **Amy S. Jordan:** Conceptualization; investigation; funding acquisition; writing – review and editing; project administration; supervision; resources; methodology; validation; visualization.

## CONFLICT OF INTEREST STATEMENT

SR was funded by the University of Melbourne (Melbourne Research Scholarship) doctoral studentship. NEB was supported by the Australian Research Council FT230100235.

## Supporting information


**DATA S1.** Supporting Information.

## Data Availability

The data that support the findings of this study are available from the corresponding author upon reasonable request.
