# Supplementary material for: Chronic sleep deficiency and its impact on pain perception in healthy females
Source: J Sleep Res. 2024 Jul 7;34(1):e14284. doi: 10.1111/jsr.14284 (PMC11744235; doi:10.1111/jsr.14284)
Supplement: Supplementary file 1 — DATA S1. Supporting Information. [file JSR-34-e14284-s001.docx]

[***Table S1.* Descriptive data (n = 41).** 1](#_Toc160726375)

[Table S2. Descriptive data for pain testing sessions Monday vs Friday (*n* = 41). 3](#_Toc160726376)

[**Table S3. Objective sleep parameters across the week in longer vs. short sleepers** 3](#_Toc160726377)

[**Table S4. Subjective sleep parameters** 5](#_Toc160726378)

[***Table S5.* Sleep characteristics and t-test for sleep paradigms in both Monday and Friday in longer vs. short sleepers** 6](#_Toc160726379)

[Figure S1. TST across the week in longer sleepers and short sleepers 9](#_Toc160726380)

[Figure S2. NREM sleep stages in longer vs. short sleepers 10](#_Toc160726381)

[**Figure S3. SOL across the week in longer vs. short sleepers** 13](#_Toc160726382)

[**Figure S4. Wake duration across the week in longer sleepers vs. short sleepers** 16](#_Toc160726383)

[Figure S5. REM duration throughout the week in longer sleepers and short sleepers 18](#_Toc160726384)

[Figure S6. Number of awakenings throughout the week in longer sleepers and short sleepers 20](#_Toc160726385)

[**Figure S7. Heat, cold, and pressure pain thresholds in longer vs. short sleepers** 23](#_Toc160726386)

[Figure S8. Temporal summation from the first to the last given painful stimuli in short vs. longer sleepers. 24](#_Toc160726387)

# ***Table S1.* Descriptive data (n = 41).**

|  | Baseline |
| --- | --- |
| Age | |
| Longer  Short | 24.8(5.3)  23.1(3.9) |
| BMI | |
| Longer  Short | 23.4(2.3)  23(5.8) |
| Racial background | |
| Asian  White  Black/African American | 29 (70.7%)  8(19.5%)  4(9.8%) |
| ESS | |
| Longer  Short | 9 (6)  9.5(4) |
| PSQI | |
| Subjective sleep quality  Longer  Short  Sleep latency  Longer  Short  Sleep duration  Longer  Short  Habitual sleep efficiency  Longer  Short  Sleep disturbance  Longer  Short  Sleeping medication  Longer  Short  Daytime dysfunction  Longer  Short  Overall  Long  Short | 1.56 (.89)  1.54(.72)  1.3(.87)  .96(.75)  1.06(.92)  1.52(.77)  .69(1)  .80(1)  3.13 (4.1)  2.7(2.3)  .19(.5)  .4(.76)  1.56(1.3)  2.12(1.1)  7(3.5)  8.24(2.7) |
| BDI | |
| Longer  Short | 9.56(6.9)  11.56(8.8) |

Table S1. Notes. Results are presented as M (SD). BDI: Beck depression index; BMI: Body mass index; ESS: Epworth Sleepiness Scale; PSQI: Pittsburgh sleep quality index.

# Table S2. Descriptive data for pain testing sessions Monday vs Friday (*n* = 41).

|  | Monday | | Friday | |
| --- | --- | --- | --- | --- |
|  | **Longer** | **Short** | **Longer** | **Short** |
| First pain testing session n (%) | | | | |
|  | 5(31.3) | 15(60%) | 11(68.8%) | 10(40%) |

# **Table S3. Objective sleep parameters across the week in longer vs. short sleepers**

|  | Sat | Sun | Mon | Tue | | Wed | | Thr | Fri | Within-Group Weekly Variation | |
| --- | --- | --- | --- | --- | --- | --- | --- | --- | --- | --- | --- |
| TST*  Longer  Short | 436.8(58.3)  285.7(80.9) | 421.2(91.2)  305.3(76.4) | 388.7(63.7)  282.7(76.2) | 450.1(55.9)  305.5(76) | 417.1(71.4)  313(63.9) | | 420.4(64)  297.4(75.4) | | 395.1(77.5)  295.9(83) | ns  ns |  |
| SOL  Longer  Short | 24.3(23)  22.3(27) | 29.1(36.5)  15(13) | 17.6(12)  21 (23.5) | 33.1(24.7)  24.3(20.2) | 33.1(35.6)  21.1(17.2) | | 43.7(56)  18.7(17) | | 29.4(18.1)  19(13.2) | ns  ns |  |
| Sleep Efficiency  Longer  Short | 91.5(4)  89.8(9.6) | 88.2(9.6)  90.1(10.5) | 92(3.8)  89.1(11.3) | 90(5.2)  90.1(5) | 88.4(7.3)  90.5(6.7) | | 87.3(9.3)  89.7(8.2) | | 90.3(4.1)  88.1(12) | ns  ns |  |
| WASO^*^  Longer  Short | 16.6(7.3)  8(4.5) | 20.6(19)  8.8(6.6) | 17(21.2)  12.6(20.2) | 14.8(5.7)  10.6(8.2) | 20.5(14.2)  11.2(12.4) | | 14.4(6.9)  9.8(9.4) | | 12(6.5)  10.9(15.2) | ns  ns |  |
| Nb. of Awakening^*^  Longer  Short | 21.3(8.4)  11.4(4.1) | 20.1(8.7)  12(4.5) | 17.7(7.1)  11.8(4.8) | 19.9(7.6)  13.4(5.8) | 21.4(11)  12.4(5.9) | | 20.3(8.2)  11(6.3) | | 17.3(8)  11(4.1) | ns  ns |  |
| Wake duration^*^  Longer  Short | 43.8(25.5)  30.9(28.9) | 53.5(34.5)  34.3(40.3) | 36.1(20.7)  33.9(32.7) | 52.1(31)  34.4(20.4) | 57.1(37.4)  34.4(25.1) | | 65.5(54.8)  33.1(23.6) | | 42.5(17.5)  38.4(38.8) | ns  ns |  |
| Bedtime^*^  Longer  Short | 01:20 (.44)  02:05(.34) | 00:36(.56)  01.34(.53) | 00:02 (.45)  01:22(.35) | 11:56(.52)  01:32(.41) | 11:29 (.44)  01:28(.34) | | 00:11(.42)  01:41(.33) | | 00:53(.50)  01:25(.39) | ns  ns |  |
| Wake-up time^*^  Longer  Short | 9:06(.51)  6.39(.38) | 8:42(.59)  6:58(.44) | 7:31(.54)  6:16(.40) | 8:57(.53)  7:37(.40) | 8:25(.47)  7:12(.35) | | 8:12(.51)  6:57(.38) | | 8(43)  6:40(.33) | *****  ns |  |

**Sleep Stage Duration**

| N1 duration ^*^  Longer  Short | 20(6.9)  13.6(3.8) | 20.6(6.8)  13.2(5.4) | 19.2(6.9)  14.8(7.4) | 21.9(5.2)  14.3(6.1) | 24.8(11)  14.6(7.3) | 21.3(7.6)  12.7(6) | 20.5(10.1)  13(5) | ns  ns |
| --- | --- | --- | --- | --- | --- | --- | --- | --- |
| N2 duration^*^  Longer  Short | 203.6(45.8)  133.7(65.4) | 173.1(45.8)  121.2(41.5) | 170.3(46)  124.9(47.1) | 207.2(50.4)  134.7(41.8) | 193.9(56.1)  131.8(54.3) | 197.3(44.4)  119.8(39.3) | 173.5(56.5)  124.7(46.4) | *****  ns |
| N3 duration^*^  Longer  Short | 91.4(27.9)  77(29.2) | 101.5(25.7)  89.2(38.7) | 99.7(19.4)  78.1(41.3) | 96.2(25)  76.6(37.2) | 96.2(21.4)  89.2(35) | 97.7(25.3)  80.2(34.1) | 101.2(23.2)  81.6(34) | ns  ns |
| NREM duration^*^  Longer  Short | 315.1(47.7)  224.4(71.5) | 295(60.1)  223.6(60.4) | 289.3(53.8)  217.9(60.8) | 325.5(51.1)  225.6(52.8) | 315(64.6)  235.7(56.2) | 316.5(54.9)  212.8(55.3) | 295.2(55.7)  219.3(59.7) | ns  ns |
| REM duration^*^  Longer  Short | 121.7(22.5)  61.2(27.2) | 125.9(42.3)  81.6(45.2) | 99.3(29.8)  68.3(37.3) | 124.7(31.6)  79.8(34) | 102.1(33.4)  77.3(35.2) | 103.9(24.8)  84.5(51.1) | 99.8(33.6)  76.5(50.7) | ns  ns |
| Wake duration^*^  Longer  Short | 43.8(25.5)  30.9(28.9) | 53.5(34.5)  34.3(40.3) | 36.1(20.7)  33.9(32.7) | 52.1(31)  34.4(20.4) | 57.1(37.4)  34.4(25.1) | 65.5(54.8)  33.1(23.6) | 42.5(17.5)  38.4(38.8) | ns  ns |

**Table S3.** Notes: Results are presented as *M* (*SD*); Within-group weekly sleep variation showed the day of the week effect in each group.

# **Table S4. Subjective sleep parameters**

|  | Sat | Sun | Mon | Tue | Wed | Thr | Fri | Within-Group Weekly Variation |
| --- | --- | --- | --- | --- | --- | --- | --- | --- |
| SOL  Longer  Short | 20(19.3)  20.3(12.6) | 19.5 (16)  24.2(31) | 18.9 (13.8)  29.9 (42) | 22.8(17.3)  21(15.4) | 23.5(16.4)  21.8(18.4) | 28.1(29.3)  15(8.8) | 24.6(28.8)  15(11) | ns  ns |
| WASO  Longer  Short | .81(1.1)  1.04(1) | .68 (1)  1.3(1,4) | .8(1.1)  1.3(1.4) | 1.5(1.7)  1(1.2) | 1.1(1.1)  1.6(1.9) | 1.1(1.3)  1 (1.6) | 1.1(1.9)  1 (1.13) | ns  ns |
| Wake duration  Longer  Short | 5.12(8.4)  4(5.3) | 4(7.9)  10(26) | 2.5(4.1)  6.2(12.6) | 5.3(14.6)  4.6(8.3) | 8.1(22.3)  12.7(27.9) | 4.4(8.4)  4.9(9.9) | 7.7(22.2)  5(9.6) | ns  ns |
| Bedtime*  Longer  Short | 01:33 AM (.48)  02.12 AM (.38) | 01:29 AM (.59)  01.38 AM (.47) | 00:46 AM (.48)  01:23AM (.38) | 00:51AM (.55)  01:28 AM (.44) | 00:36 AM (.49)  01:31 AM (.39) | 01:15 AM (.50)  01:38 AM (.40) | 01:11AM (.47)  01:26 AM (.37) | ns  ns |
| Wake-up time^*^  Longer  Short | 8:16 AM (1.9)  6:02AM (2.6) | 8:44 AM (1.9)  7:06 AM (2.2) | 7:18 AM (1.7)  6:07 AM (1.8) | 8:10 AM (2.4)  7:16 AM (1.9) | 7:56 AM (2.3)  7:24 AM (1.8) | 8:02 AM (2.3)  6:53 AM (2) | 7:11 AM (1.7)  6:58 AM (2.1) | *****  ***** |
| Sleep quality  Longer  Short | 6.12(1.3)  5.56(2.3) | 6.4(1.8)  5.2(2.4) | 6.4(1.4)  4.5(2.8) | 5.9(2.2)  5.2(2.3) | 6.5(1.6)  5.6(2.3) | 7(1.4)  5.6(1.9) | 5.3(2.1)  4.7(2.7) | ***** |
| Perceived Alert, Mood, and Fatigue | | | | | | | | |
| Alert  Longer  Short | 6.3(1.6)  5.3(2.1) | 6(2.1)  5.1(2.1) | 6(1.9)  4.9(2.2) | 6.3(1.5)  5.4(2) | 6.6.(1.8)  5.6(1.8) | 6.3(1.9)  5.6(1.6) | 5.2(2.1)  5.4(2.2) | ns  ns |
| Mood  Longer  Short | 6.3(1.3)  5.9(2) | 6.5(1.7)  5.8(2.5) | 5.8(1.9)  5.5(2.1) | 6.1(1.9)  5.9(1.7) | 6.6(1.2)  5.7(1.8) | 6.1(2)  5.7(1.9) | 6.1(1.8)  5.8(2.3) | ns  ns |
| Fatigue  Longer  Short | 11(1.7)  10.2(1.4) | 9.1(1.7)  7.8(1.4) | 8.3(2)  11.6(1.6) | 9.6(1.8)  9.3(1.5) | 9.1(1.8)  8.6(1.5) | 8.3(1.8)  8.7(1.5) | 12(1.8)  8.9(1.5) | ns  ***** |

**Table S4.** Results are presented as *M* (*SD*); alert, mood, and fatigue ranging from 0 to 10 and higher score in alert and mood means better but the opposite in fatigue.

# Figure S1. TST across the week in longer sleepers and short sleepers





Figure S1. Estimated marginal means and standard errors for total sleep time across the week between longer and short sleepers (solid and dashed lines respectively).

# Figure S2. NREM sleep stages in longer vs. short sleepers





Figure S2. Estimated marginal means and standard errors for sleep parameters across the week. Panel A to D represent N1 to NREM sleep stage in minutes.

# **Figure S3. SOL across the week in longer vs. short sleepers**





Figure S3. Estimated marginal means and standard errors for sleep onset latency across the week between longer and short sleepers (solid and dashed lines respectively).

# **Figure S4. Wake duration across the week in longer sleepers vs. short sleepers**





Figure S4. Estimated marginal means and standard errors for wake duration after sleep onset in both longer and short sleepers (solid and dashed lines respectively)

# Figure S5. REM duration throughout the week in longer sleepers and short sleepers





Figure S5. Estimated marginal means and standard errors for REM sleep in both longer and short sleepers (solid and dashed lines respectively)

# Figure S6. Number of awakenings throughout the week in longer sleepers and short sleepers





Figure S6. Estimated marginal means and standard errors for number of awakenings after sleep onset in both longer and short sleepers (solid and dashed lines respectively)

# **Figure S7. Heat, cold, and pressure pain thresholds in longer vs. short sleepers**





***Figure S7.*** Notes: Results are presented as M (SD err), Longer sleepers and short sleepers are represented in solid and dashed lines, respectively. Panel A to D represent various types of pain thresholds.

# Figure S8. Temporal summation from the first to the last given painful stimuli in short vs. longer sleepers.





Figure S8. Means and standard errors for temporal summation on Monday and Friday in longer vs short sleepers (sold and dashed lines respectively). Note: in the figure, y-axis for pain rating given to painful stimuli on scale from 0 to 10 with x-xis represents the initial (15 seconds) and final (55 seconds) pain ratings during heat painful stimuli. Both testing sessions displayed consistent initial heat pain thresholds (HPT), but a noticeable difference between the two groups emerged over time on both pain testing sessions. Furthermore, it is noteworthy that each one-unit increase in pain rating corresponds to a 1-degree decrease in pain threshold.

# Figure S9. Fatigue level throughout the week in short vs. longer sleepers.





Figure S9. Means and standard errors for the fatigue level throughout the week in longer vs short sleepers (sold and dashed lines respectively).
